# Acute nutritional ketosis: implications for exercise performance and metabolism

**DOI:** 10.1186/2046-7648-3-17

**Published:** 2014-10-29

**Authors:** Pete J Cox, Kieran Clarke

**Affiliations:** 1 Department of Physiology, Anatomy and Genetics, University of Oxford, Sherrington Building, Parks Road, Oxford OX1 3PT, UK; 2 Department of Cardiovascular Medicine, University of Oxford, Oxford, UK

**Keywords:** Ketone body, Ketosis, Ketone ester, Exercise performance, Nutrition

## Abstract

Ketone bodies acetoacetate (AcAc) and D-β-hydroxybutyrate (βHB) may provide an alternative carbon source to fuel exercise when delivered acutely in nutritional form. The metabolic actions of ketone bodies are based on sound evolutionary principles to prolong survival during caloric deprivation. By harnessing the potential of these metabolic actions during exercise, athletic performance could be influenced, providing a useful model for the application of ketosis in therapeutic conditions. This article examines the energetic implications of ketone body utilisation with particular reference to exercise metabolism and substrate energetics.

## Review

### Dietary intake influences metabolism

An ancient Spanish proverb *‘Diet cures more than the lancet’* suggests that the importance of diet in maintaining good health is an age-old concept. Mechanisms by which the body uses the fuels we eat to sustain life, or in the case of excess, store the surplus energy, have fascinated generations of scientists. Carbohydrates, fat, protein and, for some, alcohol are the fundamental sources of dietary energy. Whilst the numbers of dietary macronutrients (food groups) are limited, the particular composition and relative contribution of these dietary groups to our calorific needs vary widely. Until recently, little was known of the metabolic systems that linked diet with human function. In 1937, Krebs made arguably the most important breakthrough in biochemistry [1], describing a cycle of enzymatic reactions uniting dietary fuel combustion with cellular energy provision. This final common pathway for substrate metabolism has allowed the detailed study of the flow of energy transformation (energetics) from dietary sources to the ‘energy currency’ adenosine triphosphate (ATP).

#### Exercise the litmus of metabolic performance

Over the last century, our understanding of the fundamental processes underlying human performance has expanded greatly. At the intersection of elite sport and substrate, metabolism lays the potential to investigate the processes that define the limits of human physiology.

The onset of acute exercise triggers a rapid increase in demand for substrate and oxygen (mediated via an increase in cardiac output), with metabolic rate raised up to 100-fold above resting conditions during high-intensity exercise [2]. Depending on the relative intensity of exercise, durations of physical effort may last for minutes, hours or even days, placing large metabolic, structural and cognitive demands on body systems to sustain this output. Similar changes occur in many clinical disease states with high energy requirements, elevated cardiac output and limited tissue oxygen supply, characteristic of high dependency care, surgery or medical emergencies. The functional demands of exercise can be used to quantify responses to treatment [3], or as a diagnostic tool to identify factors limiting physical capacity [3,4]. Exercise therefore provides an ideal tool for the examination of human physical capacity and its controlling factors, under reproducible conditions.

#### High-performance athletes as models of fuel metabolism

In many respects, some of the most refined examples of human physiology and metabolism are to be found in the highly trained, athletic cohorts of competitive sport. In particular, endurance sports involving great muscular recruitment and high levels of aerobic fitness induce mitochondrial [5,6] and muscular adaptations [7-9] making such athletes ideal to study fuel metabolism. Aerobic endurance training increases mitochondrial oxidative capacity and increases selection of fatty acids for fuel at a given exercise intensity [10-12]. Increased expression of proteins involved in plasma membrane transport [13-16], and β-oxidation of fats are also well described in athletic cohorts [17]. Similarly, increases in the capacity of skeletal muscle to oxidise other blood-borne substrates such as ketone bodies have been demonstrated following athletic training [18,19]. Athletes therefore represent a useful model to further examine fuel metabolism, with predictable responses to exercise stress [12], and a well-characterised metabolic phenotype [20].

### Ketone body production: the evolutionary response to energy crisis

Ketone bodies are fuels endogenously produced by the body from mobilised fat in response to a variety of physiological [21] or pathological conditions [22]. Ketone bodies, acetoacetate (AcAc) and D-β-hydroxybutyrate (βHB), are respiratory fuels that can be oxidised by most body tissues [21] and are formed in large quantities (up to 150 g/day) by the liver in response to low blood glucose and insulin [23,24].

Ketogenesis is an evolutionary adaptation conserved within all higher order organisms to sustain survival during famine, illness or energetic stress [25]. In particular, the capacity to survive for long periods on endogenous fuel reserves is a trait of particular importance to humans where our relatively large brain size renders a steady supply of glucose critical for cerebral function. In a series of experiments in the 1960s, Cahill demonstrated the importance of cerebral ketone body oxidation in starvation, where up to 60% of the brain energy needs are derived from ketones, replacing glucose as its primary fuel [26-28]. In turn, the ketone-compensated reduction in glucose utilisation rate, and preservation of gluconeogenic protein stores [29], enables a profound increase in the capacity for survival [27]. The evolutionary effect of ketone bodies is therefore to spare carbohydrate reserves, and muscle protein, whilst themselves being an alternative energy source.

#### Exercise parallels starvation metabolism

Clearly, the protracted demands of starvation physiology occur on a much accelerated scale during sustained endurance exercise. Both conditions place a premium on glucose supply, with the finite concentrations of muscular glycogen known to be a strong determinant of exercise tolerance [30,31]. Therefore, ketosis as an evolutionary adaptation to conserve carbohydrates may provide an alternative energy substrate to power working muscle, in turn sparing intramuscular fuels. However, the application of ketone body metabolism in this context has not been appreciated.

Historically, nutritional strategies to acutely influence fuel selection during heavy aerobic exercise have largely failed to ‘spare’ muscular glycogen [32]. The reason for this seems to be that competition between substrates for respiration by working muscle is highly selective, rigidly favouring carbohydrate as relative workloads increase, despite alternative substrate provision [33,34] (see later section on fuel selection in exercise). However, as muscle carbohydrate content falls during exhaustive exercise, muscle oxidation of fatty acids does increases with a fall in respiratory quotient (RQ) [35]. A concomitant increase in blood ketone concentration is also observed, with circulating βHB levels of 1–2 mM seen following exhaustive exercise [36,37], in contrast to post-absorptive ketosis of ~0.1 mM [37]. It seems that our evolutionary response to energy crisis is hardwired to favour ketosis, and endurance exercise performance may be constrained by the same metabolic considerations pertinent to the starvation condition.

### Dietary substrates alter mitochondrial fuel preference

Randle and colleagues described the glucose-free fatty acid (FFA) cycle in 1963, suggesting an overall substrate hierarchy dominated by fatty acid selection in preference to carbohydrate for oxidative phosphorylation [38]. The capacity of the mitochondria to alter its preferential fuel selection was initially recognised by the apparent decrease in glycolysis in the presence of increased FFA. Randle suggested that carbohydrate oxidation could be regulated by the fatty acid-induced suppression of the pyruvate dehydrogenase complex (PDC) activation by a high acetyl-CoA/CoA or nicotinamide adenine dinucleotide (NADH)/NAD^+^ ratio. This in turn elevates citrate concentrations, inhibiting glycolytic flux at the level of phosphofructokinase. Whilst generally speaking, the Randle cycle approximates substrate hierarchy accurately at rest, the same is not necessarily true when cellular conditions change, such as during exercise.

### Dietary substrates and muscle fuel selection during exercise

The cellular mechanisms that control mitochondrial preference for substrates during exercise are still to be fully understood. Recent work has proven that the classical ‘glucose-FFA’ cycle is inadequate as a model of fuel selection during heavy exercise [33,39], as working muscle becomes increasingly reliant on glycolysis to provide acetyl-CoA to the tricarboxylic acid cycle (TCA) cycle, independent of FFA availability [34]. The rigid preference by muscle for carbohydrate during exercise reinforces the importance of muscular and hepatic glycogen stores for powering sustained exercise [30,40,41]. Current nutritional practice in exercise performance advocates the exogenous supplementation of carbohydrates to maintain glycaemia [42], with growing evidence to support a performance-enhancing effect during an exercise longer than 1 h [43,44] (for reviews, see [45-47]). Numerous studies have investigated dietary and pharmacological strategies to increase the exogenous provision of carbon units to the TCA cycle during exercise. Conflicting reports over the benefits of raising circulating fatty acids to spare glucose metabolism [48-52] and the failure of many studies to show convincing benefits of carbohydrate feeding [53-58] make a unifying hypothesis regarding optimal dietary strategy for performance difficult [59].

However, the nutritional provision of ketone bodies as an alternative fuel substrate may well provide a powerful signal to reinstate ‘Randle cycle’ competition between substrates for oxidative respiration [60,61]. Current literature on dietary fuel selection mechanisms has not considered the role of ketone bodies as a major fuel source in great depth, although the latter are well known to be metabolised by skeletal muscle [21]. Ketone bodies have a similar RQ to that of glucose (AcAc =1.0, βHB =0.89) if completely oxidised [62] and do not rely on glucose transporter (GLUT) or fatty acid transporters to enter cytosolic or mitochondrial spaces [63], unlike carbohydrates or fat.

Previous evidence on the role of ketone bodies to fuel muscular work in humans have been confounded by the inability to elevate ketone concentrations without the effects of starvation [64,65] or elevated fatty acids [66]. This lack of facility to induce acute ketosis has meant that all of the published literature methods to study fuel selection during ketosis have employed infusions of either AcAc or βHB in order to study the role of ketone bodies as oxidative substrates, or signals, in human subjects [67]. Narrowing this search further to those studies conducted during whole body exercise in healthy subjects results in only a handful of published reports, derived from fewer than 30 people [68,69]. None of this work studied strenuous exercise, concomitant muscle metabolism or performed work in athletic cohorts. These early investigations sought to determine the regulation of ketogenesis and its role in starvation, obesity and diabetes [70]. However, their findings leave many questions unanswered. Firstly, one of the most significant findings by Fery and Balasse et al. was a profound stimulation of exercise on the metabolic clearance of ketone bodies in overnight fasted subjects [69,71]. In two further studies, subjects performed relatively low-intensity exercise (40%–50% VO_2 Max_) for 30 min and 2 h, respectively, during constant infusions of either acetoacetate or βHB [69,72]. Circulating ketone bodies fell by >1 mM, rates of disappearance of ketones markedly increased and metabolic clearance of ketone increased approximately five- to eightfold above resting conditions. Furthermore, the percent of CO_2_ derived from the oxidation of ketones was consistently between 10.1% and 17.6% of total CO_2_, suggesting significant oxidation of ketones in overnight fasted subjects, even at relatively low workloads. This may have an important contribution to energy expenditure, thereby conserving whole body glucose stores during exercise, in addition to altering mitochondrial fuel selection and energetics, both important determinants of physical performance.

### Thermodynamics of muscle metabolism as determinants of oxidative performance

#### Conservation of energy and mitochondrial fuel selection

The energy currency ATP is required to power all mammalian cells. Cells derive *most* of their chemical energy from the combustion of carbon substrates using oxygen (although some specialised cells rely solely on anaerobic energy production). This highly regulated process occurs within the mitochondria, specialised organelles sensitive to the changing energy requirements of the cell. During exercise, ATP demand increases dramatically, placing great pressure on mitochondrial oxidative metabolism. Manipulating diet, and therefore substrate physiology, unquestionably alters human performance, and although poorly acknowledged, the reasons for these effects may lie in the thermodynamic relationships at the core of mitochondrial oxidation.

In simple terms, our body is driven by a series of controlled chemical reactions, resulting in the oxidation of carbon substrates to water and CO_2_. Thus, for a given quantity of fuel, the maximum amount of non-expansive work that can be obtained from a closed system is denoted by the Gibbs free energy (G). Described by Willard Gibbs in 1873 [73], this translation of the second law of thermodynamics relates enthalpy and entropy to the conservation of energy, expressed as:

(1) ΔG=ΔH-TΔS

Therefore, substrates with greater enthalpy can yield greater potential energy to power a system if fully oxidised. Thus, heat of combustion is of inherent importance when considering the potential impact of mitochondrial substrate selection on energetic performance (Table 1). For example, pyruvate, the end product of glycolysis, has a lower heat of combustion per C_2_ unit than either βHB or palmitate, providing less potential energy to the electron transport chain.

**Table 1:** Heat of combustion of selected fuels

**Substrate**	**∆H° kcal/mol**	**∆H° kcal/mol C**_**2 **_**units**
C_18_H_32_O_2_ (Palmitate)	-2,384.8	-298.0
C_4_H_8_O_3_ (β Hydroxybutyrate)	-487.2	-243.6
C_6_H_12_O_6_ (Glucose)	-669.9	-223.6
C_3_H_6_O_3_ (Lactate)	-326.8	-217.9
C_3_H_4_O_3_ (Pyruvate)	-278.5	-185.7

Adapted from Veech et al. [96].

From Equation 1, we can also see that the larger the value of Gibbs free energy, the more energy can be exchanged with the surrounding system. In non-standard chemical conditions, such as those encountered in human physiology or other biological conditions [74], an alternative expression of this equation is used:

(2) ΔG'=ΔG°+RTℓnQ

Therefore, by integrating the reaction quotient (Q) into its expression, Equation 2 allows the specific chemical conditions where the reaction is taking place and the principle of mass conservation to be incorporated into the calculation of free energy. Thus, Equation 2 can be further related to cellular substrate energetics, where the common endpoint for the conservation of energy arising from substrate oxidation is in the phosphate bonds of ATP. Therefore, the latent energy conserved in these bonds (∆*G*_ATP hydrolysis_) can be calculated as:

(3) $$\Delta G'\;=\;\Delta G{}^{\circ}\;+\;\ell n\;\frac{\left[ADP\right]\left[\mathrm{Pi}\right]}{\left[ATP\right]}$$

A further consideration for the application of thermodynamics in metabolism is the concept of near-equilibrium relationships between metabolic pathways, each part of a complex interdependent network, with an overall net forward flux [75]. This kinetic linkage between redox couples of the major fuel pathways and the phosphorylation potential of the cell has its origins in the early work of Haldane [76], Klingenburg [75], Krebs [77-80] and later Veech [81-85] among many others. Therefore, despite the apparent simplicity of oxidising substrates to liberate chemical energy, the useful free energy of substrate combustion to perform work is influenced by the architecture of the metabolic pathway and the enthalpy of the fuel [86]. For these reasons, the available free energy to perform work, the free energy of ATP hydrolysis (Δ*G*′_ATP_), is not equivalent for all dietary fuels.

### Mitochondrial redox state is affected by the substrate oxidised

As discussed above, the generation of the universal energy currency, ATP, requires the conversion of ADP + Pi to ATP. This process is driven by the electrochemical potential difference across the inner mitochondrial membrane. However, it should be noted that the donation of electrons to power the electron transport chain is from the reducing equivalents, NADH and flavin adenine dinucleotide (FADH_2_), both of which can be described as a redox couple with respect to the standard membrane potential (that of the hydrogen electrode, E_h_) [84]. These reducing equivalents undergo cyclical reduction and oxidation, intimately linking the TCA cycle and the electron transport chain. It becomes apparent that the larger the electrical potential difference between mitochondrial phases created by the pumping of protons into the inter-mitochondrial space [87], the greater the potential free energy. Therefore, consideration for the redox couples of the electron transport chain can be integrated into the calculation of free energy (Δ*G*′), calculated as:

(4) ΔG'=-nFΔE

(where *n* is the number of electrons and *F* is the Faraday constant). The electrochemical gradient (∆*E*) created by the electron transport chain relies on the continuous supply of reducing equivalents from NADH and FADH_2_. The energy of the proton motive force generated by the pumping of protons from the mitochondrial matrix (via complexes 1, 3 and 4 of the electron transport chain) can vary depending on the redox span between complexes of the electron transport chain [88,89]. Therefore, the relative supply of reducing equivalents generated by the architecture of each pathway influences the electrical potential difference between the (NAD^+^/NADH) couple, the co-enzyme Q couple, and thus the ΔG′_ATP_.

#### Ketone bodies alter mitochondrial energy transduction

Ketone bodies are more chemically reduced than pyruvate and result in an increased electron transport chain redox span through the reduction of the NAD^+^ couple and oxidation of the co-enzyme Q couple. This, in turn, creates a greater ∆G′_ATP_ for the generation of ATP. In the working rat heart, this has been reported to result in an increase in hydraulic efficiency (expressed as work in J/mol of O_2_ consumed) of 28% during perfusion of ketone bodies compared with glucose alone [90]. Alternative substrates such as fat are highly reduced, and thus contain a large amount of potential energy, but require more O2/mol of C2 to oxidise. In addition, the effective redox span of the mitochondrial electron transport chain is lower when fatty acids are oxidised, due to half of the reducing equivalents produced in β-oxidation being in the form of FADH2 rather than NADH. This reduces (comparatively) the redox span between (NAD^+^/NADH) couple and the co-enzyme Q couple thus reducing ∆G′_ATP_. Furthermore, elevated fatty acids induce the expression of uncoupling proteins which dissipate stored mitochondrial proton gradients and contribute to worsening metabolic efficiency through non-ATP generating metabolic cost [2].

The observed improvements in metabolic efficiency (or energetic performance) in the isolated heart may translate to greater muscular work output for a given oxygen requirement during exercise and thus sustain physical endurance. The implications of ketosis to enhance mitochondrial energetics and their potential role in disease are discussed in the detailed reviews of Veech et al. [89,91].

### Applications for ketosis to enhance athletic metabolism

Providing ketone bodies to spare intramuscular reserves mimics the physiology of starvation, where ketone bodies provide fuel for oxidation and act as signals limiting glucose and glycogen metabolism [90]. The supplementation of ketone bodies in physiologic states other than starvation may make use of our body’s hardwired metabolic response to elevated blood ketones. Ketones can be readily oxidised by the working muscle and exert a strong influence over glycolytic flux *in vivo*[21]. Elevated concentrations of ketones in a perfused working rat heart resulted in the suppression of glycolytic flux, even reporting a promotion of glycogen synthesis during continuous hydraulic work [90,92].

Ketone bodies could provide a logical alternative for the delivery of carbon units to the TCA cycle, free of the limitations in mitochondrial and sarcolemmal membrane transport that restrict fat and carbohydrate utilisation [63]. Further to acting as an alternative carbon supply, the greater enthalpy of ketone combustion over pyruvate could provide greater potential energy for conservation in the phospho-anhydridic bonds of ATP. Therefore, mimicking the physiology of starvation during exercise (by raising circulating ketone concentrations) may alter the hierarchical preference of mitochondrial substrate selection, effecting an improvement in substrate energetics.

### New frontiers in ketone metabolism

**Figure 1:**
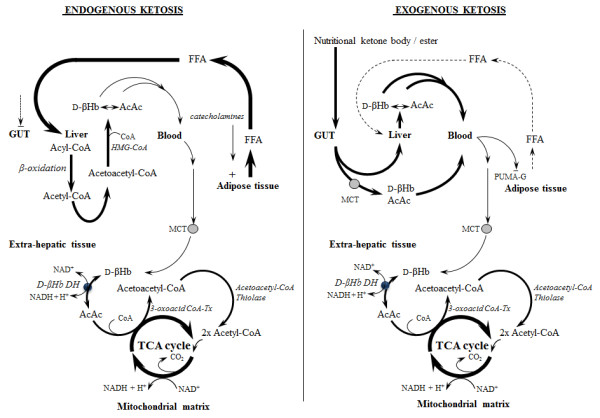
**Endogenous and exogenous ketosis.** Contrast between ketosis induced by starvation or high-fat diet (endogenous ketosis) and that generated by nutritional ketone ester ingestion (exogenous ketosis). Ketone bodies are endogenously produced in the liver from high circulating free fatty acids (FFA) from adipolysis. In contrast, nutritional ketone esters are cleaved in the gut and are absorbed through the gut epithelium and monocarboxylate transporters (MCT) into the circulation or undergo first-pass metabolism to ketone bodies in the liver. High concentrations of ketone bodies inhibit the nicotinic acid receptor (PUMA-G)-controlling adipolysis. Once released into the bloodstream, the ketones are metabolised by extrahepatic tissues in an identical fashion and being transported across the plasma and mitochondrial membranes by MCTs. D-β-Hydroxybutyrate (D-βHB) is converted to acetoacetate by D-β-hydroxybutyrate dehydrogenase (D-βHB DH) before entering the tricarboxylic acid (TCA) cycle as acetyl-CoA.

Previously, a controlled physiological ketosis required adherence to a low-carbohydrate high-fat ‘ketogenic diet’ , starvation or the administration/infusion of the salts of acetoacetate or D-β-hydroxybutyrate [93,94]. All of these methods are unpleasant, impractical or have potentially harmful side effects [95]. One possible solution to this problem is to create an ester linkage between a ketone body and an alcohol, such as 1,3-butanediol, that itself undergoes metabolism to a ketone via hepatic conversion [96]. This ester bond can be easily broken by gut or tissue esterases to release both components without the need for a salt or acid [97]. Such esters have recently been developed and tested in humans [98] and are capable of inducing the biochemical appearance of prolonged fasting within minutes of consumption. These new dietary methods to deliver a pure ketosis from exogenous sources allows, for the first time, an evaluation of ketone body metabolism itself, free of the confounding milieu required to produce ketone bodies endogenously [97] (Figure 1).

#### Ketone esters and nutritional ketosis

The first practical ketone ester synthesised to be hydrolysed in plasma, free of a sodium salt load and effectively induce a rapid circulating ketonaemia was described by Birkhahn et al. [99,100] in the late 1970s. This monoester of glycerol and acetoacetate (monoacetoacetin) was delivered parenterally to rats. Prior to this, the butyl alcohol, *RS*-1, 3-butanediol, was known to be oxidised in the liver, producing both βHB and AcAc in isolated liver mitochondria [101], rats [102] and humans [103]. Two iso-enantiomers of βHB were produced from the administration of a racemic mix of RS-1,3-butanediol, with the *S* enantiomer not naturally found within the body [101], although it is oxidised by body tissues [104-106]. In 1995, the administration of both oral and parenteral ketone esters containing RS-1,3-butanediol, and either βHB or acetoacetate, was described in pigs by Desrochers [107]. Now, human safety and tolerability trials have been successfully undertaken using ketone monoesters of βHB and R-1,3-butanediol [98]; the opportunity to examine ketosis in detail during a number of therapeutic and physiological conditions appears a step closer.

### Not all ketosis is equivalent; high-fat diets vs. exogenous ketones

The popularity of ketosis as a weight loss intervention by adherence to a high-fat, low-carbohydrate diet (for systematic review, see [108]) owes much of its notoriety to the Atkin’s diet fad of the early 2000s [109]. However, ketogenic diets are far from a novel discovery. The Inuits had almost exclusive intake of dietary fat and protein throughout the long arctic winter, where naturally occurring dietary carbohydrate sources are virtually non-existent. However, the metabolic conditions of chronic dietary ketosis are in stark contrast to the rapid exogenous delivery of ketone bodies now possible with ketone esters. In essence, the efficacy of the low-carbohydrate diet is dependent upon depleted hepatic and muscle carbohydrate reserves increasing circulating FFA and endogenous ketone body production. Low muscular carbohydrate content during heavy sustained exercise is well known to impair physical performance [30]. The recent interest in low-carbohydrate diets to enhance submaximal exercise tolerance [110-112] are not thought to be driven by ketosis, rather by an up-regulation in fatty acid oxidation [51] (in lieu of low muscle glycogen content) over weeks of specific dietary intervention [113]. Acute exogenous delivery of ketone bodies elevates ketone levels without the prior depletion of muscle carbohydrates necessary to induce ketosis via endogenous production.

## Conclusion

Ketone bodies have long been overlooked as alternative substrates to power our bodies. The reasons for this are numerous but in no small part related to the negative connotations associated with the discovery of ketosis in critically ill diabetic patients [22,114]. Furthermore, ketosis has until now only been achievable in starvation states or high-fat low-carbohydrate diets, conditions which are unpleasant, difficult to sustain and negate many of the desirable effects of ketone metabolism [115]. The evolutionary conservation of ketone bodies as energy substrates has a sound rationale, their being thermodynamic advantages to their oxidation, as well as the preservation of alternative energy reserves essential to our survival. The importance of oxidative efficiency and conservation of carbohydrate reserves is vital not just in starvation but on a greatly accelerated scale during endurance exercise. Exercise places large demands on oxidative metabolism for the sustained provision of ATP to the working muscle. Finite reserves of intramuscular glycogen, and a loss of flexibility for mitochondrial fuel selection during high intensities of aerobic work [33], underline the importance of substrate metabolism for athletic performance. Given the well-characterised demands of endurance exercise and the importance of dietary substrates on athletic metabolism, there is a clear rationale for the nutritional provision of exogenous ketone bodies in this context. With the recent development of novel forms of dietary ketone ester now undergoing rodent and human testing, the stigma of this much maligned substrate may yet be challenged.

## Abbreviations

βHB: D-β-hydroxybutyrate; AcAc: acetoacetate; FFA: free fatty acids; TCA: tricarboxylic acid cycle; ATP: adenosine triphosphate; NADH: nicotinamide adenine dinucleotide; FADH_2_: flavin adenine dinucleotide; P_i_: inorganic phosphate; ADP: adenine diphosphate; RQ: respiratory quotient.

## Competing interests

Professor Kieran Clarke is a non-executive director of TdeltaS Ltd, a spin out company of the University of Oxford, which owns the intellectual property rights to the D-3-β-hydroxybutyrate-1,3-butanediol ketone monoester. Dr Peter Cox declares that he has no competing interests.

## Authors’ contributions

PC conceived the review, and both PC and KC drafted the manuscript. Both authors read and approved the final manuscript.
